# The Trauma Burden of Electric Scooter-Related Injuries Presenting to a Single-Centre Trauma and Orthopaedic Department in Central Birmingham

**DOI:** 10.7759/cureus.98978

**Published:** 2025-12-11

**Authors:** Safina Begum, Muaaz Tahir, Omar Mostafa, Divya Prakash

**Affiliations:** 1 Trauma and Orthopaedics, University Hospitals Coventry and Warwickshire NHS Trust, Coventry, GBR; 2 Trauma and Orthopaedics, Birmingham Orthopaedic Training Programme, Birmingham, GBR; 3 Trauma and Orthopaedics, Sandwell & West Birmingham NHS Trust, Birmingham, GBR

**Keywords:** electric scooters, e-scooters, orthopaedic trauma surgery, scooter injury patterns, trauma burden

## Abstract

Background and objective

Electric scooters (e-scooters) have been adopted as a popular mode of transport, both in the UK and internationally, as part of a growing movement for greener forms of transport. Despite its environmental advantages, e-scooter use has been associated with rising trauma presentations to healthcare systems worldwide. This study aimed to evaluate the burden of e-scooter-related injuries presenting to a single trauma and orthopaedic (T&O) centre before and after the introduction of the Birmingham e-scooter rental scheme.

Methods

A retrospective observational study was conducted to identify all e-scooter-related trauma presentations referred to the on-call T&O department at an inner-city hospital in Birmingham between 2018 and 2022. Data collected included patient demographics, injury patterns, surgical intervention rates, and length of hospital stay. Injuries were classified into long-bone fractures, other fractures, soft tissue injuries, spinal injuries, and head injuries. The study included both e-scooter riders and pedestrians.

Results

A total of 74 patients were included in the study, with a median age of 21 years (range: 2-86), and 73% (n = 54) were male. Limb fractures accounted for 73% (n = 54) of injuries, including 50% (n = 27) long-bone fractures and 11% (n = 6) open fractures. Soft tissue injuries comprised 19% (n = 14) of total injuries, head injuries 7% (n = 5), and spinal injuries 1% (n = 1). Of note, 70% of all patients required surgery for their injuries, with a mean inpatient stay of 3.4 days, and four patients were identified as pedestrians. The majority of the e-scooter-related trauma (87.8%) occurred after the introduction of the rental scheme in October 2020.

Conclusions

The introduction of the Birmingham rental e-scooter scheme has been associated with a marked increase in orthopaedic trauma, with a significant proportion requiring operative management. This highlights the significant burden of e-scooter-related trauma on orthopaedic services and emphasises the need for enhanced safety measures and evaluation of the impact of such schemes on healthcare services.

## Introduction

Electric scooters (e-scooters) have rapidly emerged as a popular mode of transport worldwide, particularly among younger populations seeking a convenient and affordable alternative to traditional transportation. In June 2020, the Department of Transport launched an e-scooter rental trial, accelerated by the COVID-19 pandemic, as part of a UK-wide initiative to promote alternative modes of travel. The rental schemes have been trialed in 33 UK cities, with Birmingham being the first to implement the program in October 2020. Initiatives behind the rental schemes included promoting sustainable and environmentally friendly forms of transport, which are both cheaper and more convenient for users [1].

Despite their advantages, the rising popularity of e-scooters and the rollout of rental schemes in the UK and globally have raised significant safety concerns for both riders and pedestrians. The current operational requirements set by the Department of Transport for the national rental e-scooter schemes restrict use to roads and cycle lanes, impose a maximum speed limit of 15.5 mph, and require riders to be at least 16 years old with a full or provisional driving licence, while helmet use remains optional [2]. Healthcare services worldwide have reported a substantial increase in e-scooter-related trauma presenting to emergency and orthopaedic services [3-5], with national reports indicating that since 2020, over 1,900 casualties have been reported in e-scooter collisions, alongside 34 fatal cases [6].

This single-centre retrospective observational study aimed to assess the burden of trauma associated with e-scooter use by examining patient demographics, injury patterns, and their clinical management. Although several studies have demonstrated a rising burden of trauma associated with e-scooter use, limited evidence exists on how national rental schemes have influenced this trend. This study, therefore, aims to evaluate the impact of the Birmingham rental scheme on the pattern and severity of e-scooter-related injuries presenting to a single trauma centre. By characterising the impact of e-scooter-related trauma, the study seeks to provide evidence to inform improved safety measures and guide future regulatory policies.

This article was previously presented as an abstract at the 2023 ASIT meeting on March 4, 2023, and subsequently published in the British Journal of Surgery as an abstract - abstract citation ID: znad258.673.

## Materials and methods

This retrospective observational study was conducted in a trauma and orthopaedic (T&O) Department at a single secondary-care centre in central Birmingham. All data were collected retrospectively. The hospital’s T&O on-call trauma lists, maintained in Microsoft Excel, were systematically reviewed to identify all patients presenting with electric scooter-related injuries during the study period. The review covered four years, from 2nd August 2018 to 19th December 2022. The incidence of e-scooter injuries was compared for the two years before and the two years after the launch of the Birmingham e-scooter rental scheme in October 2020, in order to evaluate any change in the trauma burden following its introduction.

Trauma take lists were screened to identify any entries containing the following keywords: “electric”, “e”, and/or “scooter”. Identified cases were then manually reviewed to confirm relevance and ensure inclusion criteria were met. Non-electric scooters, including mobility scooters and push scooters, were excluded from this study, as only injuries resulting specifically from electric scooter use were included.

For each confirmed electric scooter-related presentation, patient records and referral details were examined to determine the nature of the injury and subsequent clinical management. Injuries were categorised into predefined groups: soft-tissue injuries, long-bone fractures, other fractures (including ankle, hand, and facial fractures), spinal injuries, and head injuries. Additional clinical variables were extracted, including whether operative intervention was required, the presence of an open fracture, the length of inpatient hospital stay, and whether the patient sustained polytrauma as a result of the injury. The study included electric scooter-related injuries sustained either by riders or by pedestrians involved in e-scooter incidents.

Inclusion criteria comprised all acute referrals and presentations to the T&O service with confirmed electric scooter-related injuries during the study period. Exclusion criteria included injuries related to non-electric scooters, such as mobility scooters and push scooters.

## Results

A total of 74 patients were referred to the on-call T&O service with electric scooter-related injuries during the study period (Table 1). The ages of the patients ranged from two to 86 years, with a median age of 21 years. The majority of cases were male (73%, n = 54), while females comprised 27% (n = 20). Four patients (5.4%) were pedestrians who sustained injuries as a result of e-scooter collisions.

**Table 1:** Demographics of patients presenting with electric scooter–related injuries between August 2018 and December 2022 (n = 74)

Variables	Values
Male, n (%)	54 (73)
Female, n (%)	20 (27)
Median age (range)	21 (2-86)
Rider, n (%)	70 (94.6)
Pedestrian, n (%)	4 (5.4)

Most of the presentations involved orthopaedic trauma, with 73% (n = 54) sustaining limb fractures. Among these, 50% (n = 27) were long-bone fractures, and 11% (n = six) were open fractures, reflecting a substantial proportion of higher-energy mechanisms. Other injuries included soft-tissue trauma in 19% of patients (n = 14), head injuries in 7% (n = five), and one case of a spinal injury (1%). Regarding clinical management, 70% of all patients required operative intervention. Among those treated surgically, the mean inpatient stay was 3.4 days, indicating a measurable impact on hospital bed utilisation and postoperative care resources. 

**Figure 1:**
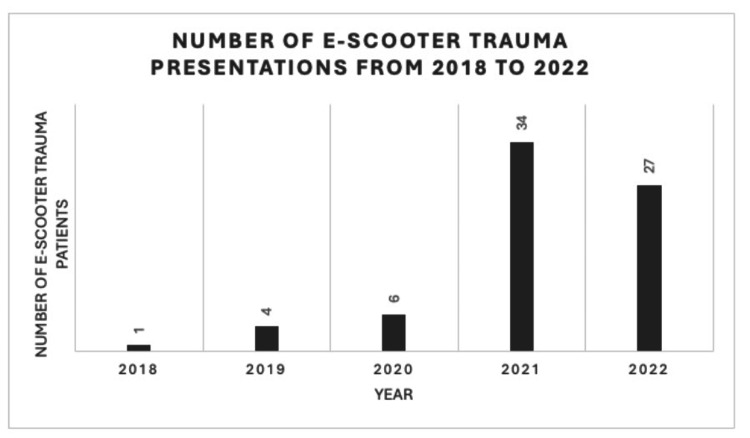
Annual cases of electric scooter-related trauma presentations from 2018 to 2022 The graph shows a marked increase following the introduction of the Birmingham e-scooter rental scheme in October 2020

A clear trend was observed over the study period (Figure 1). The number of annual presentations of e-scooter-related trauma increased from one case in 2018 to four in 2019 and six in 2020. This was followed by a substantial rise to 34 cases in 2021 and 27 cases in 2022. Overall, 87.8% of all e-scooter injuries occurred after the introduction of the Birmingham e-scooter rental scheme in October 2020, indicating a strong temporal association between increased availability of rental e-scooters and the growing incidence of trauma presentations.

## Discussion

The main finding of this study was the significant rise in e-scooter-related trauma following the introduction of the rental scheme in central Birmingham. E-scooter collisions led to significant injuries for both riders and pedestrians, with the vast majority of injuries involving significant orthopaedic pathology. Most cases of e-scooter trauma in this study sustained limb fractures, including a significant proportion of long-bone and open fractures. The majority of patients (70%) required surgical management for their injuries, which led to an average hospital stay of 3.4 days, indicating the potential of high-energy injuries secondary to e-scooter use. Comparable operative rates have been reported elsewhere; Coelho et al. found that 49.6% of fractures sustained in e-scooter collisions required surgical management, highlighting a similarly high burden of operative orthopaedic care [4].

Similar observations have been reported in a study in New Zealand, where Brownson et al. identified a substantial proportion of e-scooter injuries requiring hospital treatment, including 22% that necessitated surgical intervention, further illustrating the significant orthopaedic burden associated with this mode of transport [3]. Other injuries observed in our cohort included soft tissue injuries, head injuries, and spinal trauma - this pattern indicates that although limb fractures were the predominant injury type, other anatomical regions are also vulnerable. These injury patterns also align with the scoping review by Toofany et al., which identified extremity injuries and head trauma as the most common presentations following e-scooter collisions [7]. Our study cohort was predominantly young and male, and although riders accounted for most cases, pedestrians were also affected.

We observed a sharp increase in presentations from 2021 onwards, with 87.8% of all cases occurring after the rental scheme was introduced. This is supported by national government statistics, which show an increase in e-scooter collisions by 196% in 2021 vs. 2020 [6]. National findings from the Parliamentary Advisory Committee for Transport (PACTS) casualty data report, similarly, demonstrate a steady rise in e-scooter-related injuries across the UK, supporting the upward trend observed in our cohort [8]. Their data highlight increasing numbers of both rider and pedestrian casualties, consistent with our identification of injuries in both groups. These findings are supported by Dela Cruz et al., who reported a three-fold increase in orthopaedic injuries related to e-scooters in central London following the announcement of the government e-scooter trial scheme [9]. A similar pattern has been reported internationally, as Shichman et al demonstrated a six-fold increase in monthly e-scooter injuries following the introduction of shared e-scooter services [10]. Their findings parallel our observation of a marked surge in cases after the Birmingham rental scheme was launched, reinforcing the association between expanding e-scooter availability and escalating trauma burden.

The mechanism of injury in our cohort was predominantly falls from the e-scooter. These results are in line with those published by Luceri et al, who reported loss of balance and subsequent falls as the primary cause of e-scooter-related orthopaedic trauma presenting to an emergency department in Italy [5]. Although our study did not assess the financial impact of e-scooter trauma, national data by PACTs reported that e-scooter injuries presenting to Emergency Departments cost the NHS nearly £1,000 per patient on average [8]. Bekhit et al. similarly highlighted significant regional healthcare expenditure associated with these injuries [11].

This study provides e-scooter trauma data over five years, highlighting patient demographics, injury patterns, operative requirements, and inpatient stay for both riders and pedestrians. However, only e-scooter injuries referred to the on-call T&O service were included, so cases managed solely by the Emergency Department or fracture clinics were not captured, potentially underestimating the true injury burden. As a single-centre, retrospective study, the findings may not be generalisable, and financial impact and long-term outcomes were not assessed. Additionally, behavioural factors such as helmet use and intoxication status were inconsistently recorded in clinical records and therefore could not be incorporated into the analysis. Omission of these variables may influence the interpretation of the mechanism of injury and risk profiles.

This study did not differentiate between rental and private e-scooter use, as this information was not routinely documented in clinical records. This distinction is further complicated by the illegality of private e-scooter use on public roads, which may discourage accurate disclosure by patients and limit the reliability of such data. Despite these limitations, the study offers valuable insights into the burden of e-scooter-related trauma following the introduction of a rental scheme.

## Conclusions

E-scooters have become an increasingly popular mode of transport, especially among younger individuals, due to their convenience and environmentally friendly nature. However, this study highlights the considerable risk of injuries associated with their use. Since the introduction of the rental e-scooter scheme, the incidence of e-scooter-related trauma has risen markedly and is likely to continue to increase as the service expands. The injuries sustained by both riders and pedestrians frequently involve significant orthopaedic trauma, with many requiring operative intervention and inpatient admission. These patterns of injury are reflective not only of the growing local burden on T&O services but also of wider national pressures on emergency care, surgical pathways, and NHS resources. This study further emphasises the clear need for a comprehensive review of e-scooter safety measures and enforcement strategies before any future legislation is implemented. Future studies should evaluate the financial consequences associated with e-scooter injuries to better appreciate the economic burden of this transport service.
